# Polyamine Metabolism in Fungi with Emphasis on Phytopathogenic Species

**DOI:** 10.1155/2012/837932

**Published:** 2012-08-22

**Authors:** Laura Valdés-Santiago, José Antonio Cervantes-Chávez, Claudia Geraldine León-Ramírez, José Ruiz-Herrera

**Affiliations:** ^1^Departamento de Ingeniería Genética, Centro de Investigación y de Estudios Avanzados del Instituto Politécnico Nacional, Unidad Irapuato, Km 9.6, Libramiento Norte, Carretera Irapuato-León, 36821 Irapuato, GTO, Mexico; ^2^Facultad de Ciencias Naturales, Universidad Autónoma de Querétaro, Anillo Vial Fray Junipero Serra Km 8 S/N, Carretera a Chichimequillas, 76000 Santiago de Querétaro, QRO, Mexico

## Abstract

Polyamines are essential metabolites present in all living organisms, and this subject has attracted the attention of researchers worldwide interested in defining their mode of action in the variable cell functions in which they are involved, from growth to development and differentiation. Although the mechanism of polyamine synthesis is almost universal, different biological groups show interesting differences in this aspect that require to be further analyzed. For these studies, fungi represent interesting models because of their characteristics and facility of analysis. During the last decades fungi have contributed to the understanding of polyamine metabolism. The use of specific inhibitors and the isolation of mutants have allowed the manipulation of the pathway providing information on its regulation. During host-fungus interaction polyamine metabolism suffers striking changes in response to infection, which requires examination. Additionally the role of polyamine transporter is getting importance because of its role in polyamine regulation. In this paper we analyze the metabolism of polyamines in fungi, and the difference of this process with other biological groups. Of particular importance is the difference of polyamine biosynthesis between fungi and plants, which makes this process an attractive target for the control of phytopathogenic fungi.

## 1. Introduction

Polyamines constitute a group of ubiquitous and essential aliphatic polycations found in both eukaryotic and prokaryotic organisms [1]. In higher eukaryotic organisms including fungi, the most common polyamines are putrescine, spermidine, and spermine; nevertheless, a large number of fungal species do not contain spermine. In general, it is accepted that the role of polyamines is to regulate several known and unknown biological processes. Polyamine depletion in the cells results in growth cessation [2, 3], whereas excessive intracellular accumulation of polyamines may be cytotoxic [4], indicating the necessity of a strict regulation of the intracellular polyamines pools.

 Addition of exogenous polyamines prolongs the life span of several organisms such as *Saccharomyces cerevisiae, Caenorhabditis elegans, *and* Drosophila melanogaster* [5]. In fungi, as occurs with the rest of living organisms, polyamines are essential to support growth, thus, mutants affected in their synthesis become auxotrophic to the missing polyamine. Additionally, they regulate a wide variety of biological phenomena including differentiation processes, for example, dimorphism, spore germination, and appressorium formation and conidiation [6, 7]. In some way or another, polyamines, regulate the virulence of animal and plant fungal pathogens. Considering the positive charge of polyamines it is not surprising that polyamines act through binding to and stabilizing polyanionic macromolecules of the cell, such as DNA, RNA, membrane phospholipids, and some cell wall components. Considering the size of polyamines, it has been demonstrated that their capacity to bind polyanions is superior to that of Mg^+2^ cations. Through this association polyamines can modulate gene expression, enzymatic activities, translation, and DNA-protein interactions [8–10]. 

It is important to mention that in plants there exist at least two pathways involved in polyamine biosynthesis; in contrast, in fungi there is a unique pathway leading to polyamine formation in which the enzyme ornithine decarboxylase plays the central role. This characteristic makes this pathway an ideal target for the control of fungal diseases in plants without any secondary effect on the polyamine metabolism of the hosts.

One important aspect to consider in the study of polyamine metabolism is whether the different polyamines play different and specific roles in the cells. This problem is more difficult to approach in organisms that possess the three polyamines described above. The use of fungi that, as occurs in *Ustilago maydis*, contain only putrescine and spermidine is a possible solution to simplify this problem. *U. maydis *is a dimorphic fungus and the causal agent of common smut or “huitlacoche” in maize; during the life cycle of *U. maydis *several different stages can be observed. These include a saprophytic form growing as budding yeast, a dikaryotic mycelium phase that is the product of the mating of two sexually compatible yeast cells. This invades the host and grows in all the aerial maize tissues. Eventually, the dikaryotic mycelium suffers morphogenetic changes that end in the production of diploid teliospores. *U. maydis *is an interesting model system for the study of polyamine metabolism because of its following characteristics: (a) its ability to complete the life cycle in a short time [11], (b) the existence of an haploid phase, and a sexual cycle [11], (c) the presence of an efficient transformation system [12, 13], (d) the existence of tools for molecular genetic analysis [14, 15], (e) the presence of differentiative phenomena affected by polyamines [16], (f) the presence of only two polyamines: putrescine and spermidine [17].

In the following pages we describe the metabolism of polyamines in fungi with emphasis on its importance in phytopathogenic fungi, including *U. maydis*.

## 2. Polyamine Metabolism in Fungi

**Figure 1:**
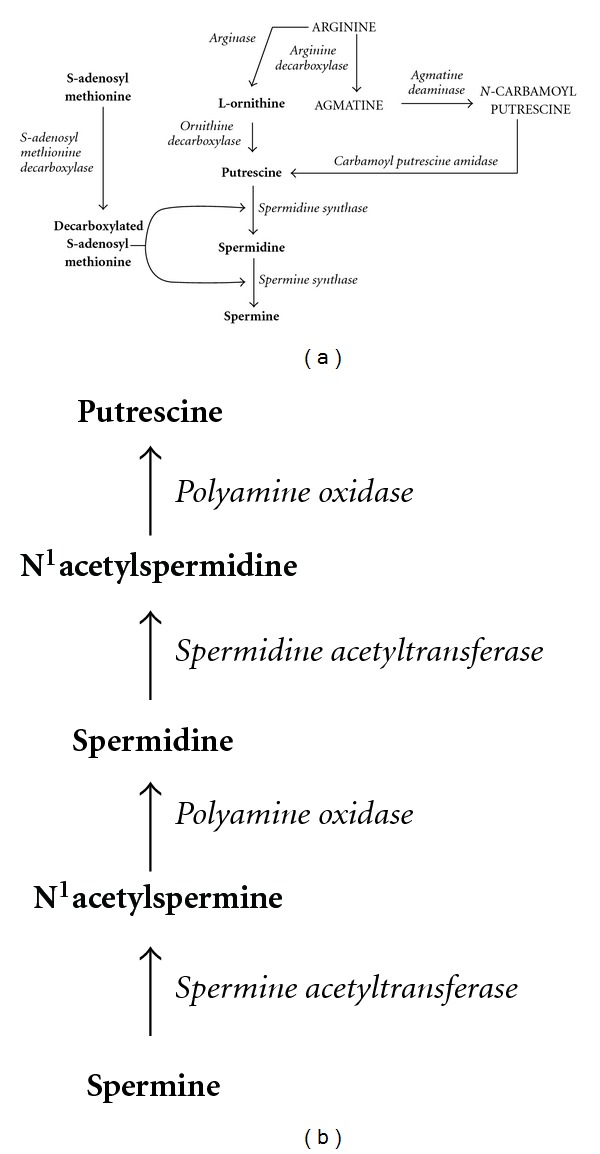
Polyamines biosynthetic pathway. (a) Comparison between biosynthetic pathways present in fungi and plants. (b) Polyamine retroconversion mechanism in fungi. Bold letters indicate products of the metabolic pathways in fungi. Italic letters indicate the enzymes that mediate the chemical reaction. Capital letters indicate products of the metabolic pathway exclusive in plants.

The biosynthetic pathway of polyamines in fungi is similar to that occurring in animals starting with ornithine that by action of the rate limiting enzyme ornithine decarboxylase (Odc) (E.C.4.1.1.17) gives rise to the intermediate diamine putrescine. Conversion of putrescine into the triamine spermidine involves the addition of an aminopropyl group by the action of the enzyme spermidine synthase (Spe) (E.C.2.5.1.16). The aminopropyl group is derived from *S-*adenosylmethionine (SAM), by the action of *S-*adenosylmethionine decarboxylase (Samdc) (E.C.4.1.1.50) that produces decarboxylated S-adenosylmethionine (dcSAM). In the final step of the polyamine pathway, spermine synthase (Sps) (E.C.2.5.1.22) transfers a second aminopropyl group to the acceptor spermidine to form the tetramine spermine. In addition, it occurs a retroconversion mechanism, where through several reactions spermine is converted into spermidine, and this is converted into putrescine [18]. The starting reaction of this pathway is an acetylation of the polyamine, a reaction catalyzed by spermidine or spermine *N* ^1^-acetyltrasferases (Ssat) (E.C. 2.3.1.57) producing *N* ^1^-acetylspermine or *N* ^1^-acetylspermidine, respectively. The reaction involves the transfer of an acetyl group from acetyl-coenzyme A to the *N* ^1^ position of either spermidine or spermine. It is also known that spermine can be diacetylated, preventing its transformation into putrescine [19, 20]. The monoacetyl derivatives undergo an oxidative splitting by the enzyme polyamine oxidase [21] (E.C. 1.5.3.11), a flavine adenine dinucleotide-dependent enzyme [21], which cleaves at the internal nitrogen to yield *N-*acetylpropionaldehyde and putrescine or spermidine depending on the substrate [22]. The biosynthetic pathways of polyamines in fungi and plants are presented on Figure 1. It has been demonstrated that not all pathogenic fungi possess all the enzymes involved in the general metabolic reactions described above.

In mammalian cells and plants, a direct mechanism of backconversion of spermine to spermidine has been suggested [23–28]. Nevertheless, in fungi there is no clear evidence about direct backconversion of spermine to spermidine or spermidine to putrescine. In 2003 Landry and Sternglanz [29] described in *S. cerevisiae *that its polyamine oxidase was able to oxidize spermine to spermidine. However, it was not tested if this reaction occurred *in vivo. *Whether any of the fungal Pao's can convert spermine to spermidine or spermidine to putrescine is something that needs to be proved by biochemical analysis. On these bases it can be concluded that, in fungi, oxidative transformation of spermine to spermidine, and spermidine to putrescine takes place only on acetylated polyamines, as we demonstrated to occur in *Ustilago maydis* [30].

 Regarding degradation of polyamines, this occurs by a different pathway. Thus, putrescine can be oxidized by a diamine oxidase yielding *γ*-aminobutyraldehyde, which can be further oxidized to *γ*-aminobutyrate (GABA), or else the Δ^1^-pyrroline (the spontaneously cyclic form of *γ*-aminobutyraldehyde) may be converted into 2-pyrrolidone and 5-hydroxy-2-pyrrolidone [31, 32]. Putrescine can also be acetylated by a microsomal enzyme and the resulting monoacetyl-putrescine then oxidized by monoamine oxidase. Most terminal polyamine catabolites are non-*α*-amino acids and *γ*-lactams [22, 33]. 

As indicated above, most of the eukaryotic organisms synthesize putrescine, spermidine, and spermine, whereas prokaryotes are unable to synthesize spermine. The exception mentioned also above is fungi that being eukaryotic organisms not all contain spermine [34]. The gene encoding *SPS* is found only in the Saccharomycotina class of the Ascomycota, which consists of the true yeasts [35].

The presence or absence of the genes involved in polyamine metabolism in phytophathogenic fungi is shown in Table 1. These data were obtained by means of a BLAST analysis using *S. cerevisiae* genes as template. We found that all phytophathogenic fungi examined contained protein homologous to *ODC* and *SPE* genes. We include three *Phytophthora* species although the genus is no longer considered to belong to kingdom Fungi, but to be a member of Chromista.

Although the metabolism of polyamines in fungi shares some similar reactions with plants, it still shows some differences that may allow us to target polyamines synthesis as a control of fungal plant diseases. In plants, putrescine, spermidine, and spermine are the most abundant polyamines, but unlike fungi, putrescine is synthesized by two alternative pathways (Figure 1), directly from ornithine by Odc as described above for fungi and animals, and by another pathway, where arginine is converted into agmatine by the action of the enzyme arginine decarboxylase (Adc) (E.C. 4.1.1.19), a reaction that is followed by additional steps to produce putrescine [36]. 

## 3. Inhibition of Polyamine Metabolism 

In the past decades polyamine metabolism in fungi has attracted the attention of researches worldwide with, among other aspects, the aim to manipulate it and use it as a strategy to control fungal plant diseases. The first consideration to study this promising aspect was the possibility to inhibit the polyamine synthesis pathway using compounds specifically targeted to key enzymes of the pathogens. The inhibitors most widely used under this approach have been D, L-*α*-difluoromethylornithine (DFMO) and 1–4 diamino butanone (DAB) [37] both of which inhibit the rate limiting enzyme Odc, in such a manner that its inhibition would be lethal to the pathogen, whereas the plant host would be able to survive using the alternative pathway involved in polyamine biosynthesis (see above). Another inhibitor, *α*-difluoromethylarginine (DFMA), inhibits Adc activity, and it has been observed that in some fungi it inhibits Odc, due to the conversion of DFMA to DFMO by the enzyme arginase, as suggested in *Botrytis cinerea*, *Phytophthora infestans*, and *Sclerotinia sclerotiorum *[38–41]. Other enzymes involved in polyamines synthesis are inhibited by some compounds like cyclohexylamine (CHA), an inhibitor of Spe, or methyglyoxal bis-[guanyl hydrazone] (MGBG) that inhibits Samdc.

 There are several fungi that are affected by this kind of inhibitors. One of them is *Colletotrichum truncatum*, a fungus able to attack soybean, one of the most important worldwide crops, where it is the causal agent of the disease known as anthracnose. When putrescine biosynthesis was inhibited by DFMO or DFMA, growth of *C. truncatum *was inhibited [42]. The same result was obtained by the use of DFMO on other economically important plant pathogens such as *Rhizoctonia solani, B. cinerea, Fusarium oxysporum, and Cochliobolus carbonum *and the fungus-related organism, the Chromista species *P. infestans *[40, 43–45].

 The soil borne plant pathogen *S. scletoriorum* is worldwide distributed threatening many crops. Addition of DFMO or DFMA to this pathogen inhibited mycelial growth [39]; and this effect was reverted by exogenous putrescine when the fungus was challenged with DFMO, however, the addition of putrescine did not induce mycelial growth when *S. scletoriorum *was treated with DFMA. The failure to revert the effect of DFMA by exogenous putrescine suggests that this compound may have exerted other toxic effects in the cell [39]. On the other hand, it was observed that during sclerotial development, a higher concentration of intracellular polyamines is necessary, considering that a very small amount of DFMO was required to inhibit this differentiation process, that was inhibited not only by DFMO, but also by DFMA and CHA [39]. Although DFMO was able to inhibit mycelial growth in this fungus, ascospore germination was not affected, neither by DFMO nor CHA, although germination was inhibited by addition of MGBG. Interestingly, its effect was not reverted by exogenous polyamines suggesting the possibility that other toxic effects were exerted by MGBG, or that polyamines have a low degree of penetration rate into ascospores [46]. Unfortunately, the disease index tested on tobacco leaves was not reduced by the use of these inhibitors, possibly taking into account that the amount of polyamines into ascospores is quite high. In addition, mycelium emerged from ascospores no matter the presence of the inhibitor applied; apparently under these conditions the mycelium acquired certain autonomy being now able to uptake polyamines from the plant [46].

In a similar way, in *Colletotrichum gloesporioides*, a pathogen of red pepper and avocado, addition of polyamines had an inhibitory effect, as observed by the degree of inhibition during the conidial germination and appressorium development. In addition, the Samdc inhibitor MGBG also affected both biological processes [47]. Interestingly, this effect was overcome by the addition of calcium, suggesting the involvement of the calmodulin/calcineurin signaling during the plant invasion process [47]. According to this hypothesis, it was observed that the *C. gloesporioides* gene encoding calmodulin (*CgCAM*) was expressed during appressorium development and that this expression was repressed by exogenous spermidine, an effect that was counteracted by the addition of calcium. These data reveal the interplay that must exist between polyamines and other signaling pathways to orchestrate specific biological phenomena in time and space.

In most cases, addition of DFMO or DFMA resulted in fungal growth reduction as occurred with *S. sclerotiorum* whose mycelial growth was effectively reduced by a 1 mM concentration of either compound. A similar effect was observed in the fungus-related Chromista species *Phytophthora sojae*, a soil borne phytopathogen, where DFMO addition also reduced its hyphal growth, this reduction, being easily reverted by the addition of exogenous polyamines. Interestingly, the zoospores of this pathogen are able to depend on their internal polyamine storage until they establish contact with the host [48].

A contrasting different scenario was observed in the most devastating rice pathogen *Magnaporthe grisea. *During conidiation of this ascomycota, the most abundant polyamine was spermidine, with only very low amount of putrescine [49]. During the germination process of conidia, no significant change in the spermine or putrescine contents was detected, but spermidine decreased rapidly along with the germination event. An interesting observation was that development of the appressorium, which is an indispensable cell structure for the fungus to get access into the plant, was inhibited by the addition of exogenous polyamines either putrescine, spermidine, or spermine, revealing in this way that polyamine metabolism must be under a fine tune regulation mechanism. In this regard, addition of DFMO or MGBG had no visible effect during development and function of the appressorium [49]. It is interesting to notice that in *M. grisea* a crosstalk between polyamine metabolism and signaling through the cAMP pathway possibly regulates appressorium development, considering the observation that elevation of the cAMP levels either by addition of exogenous cAMP or IBMX (a potent phosphodiesterase inhibitor) was able to restore appressorium development at a normal rate even in the presence of polyamines [49].

Regarding DFMO, we must recall that this chemical does not inhibit the synthesis of polyamines by plants because, as described above, plants synthesize polyamines by the arginine pathway as well [50]. Worth to mention is the fact that the high cost of DFMO makes it unsuitable to be used as a fungicide, and additionally that it is quite toxic to animals, and that the diseases caused by some plant pathogens such as *Septoria tritici*, *U. maydis, Stagonospora nodorum*, *Pyrenophora avenae*, and *Ophiostoma ulmi *[51, 52] are not relieved by this compound. This result is probably due to low permeability of the drug, although this phenomenon has been also explained in function of an alternative route for polyamine biosynthesis in these organisms considering that Adc activity has been detected in *Verticillium dahliae *[53] (an unlikely possibility), and taking into consideration that, in theory, plants contain sufficient polyamines to support fungal growth even in the presence of inhibitors of Odc to support the growth of the pathogen in the plant tissues. The effect of polyamine synthesis inhibitors is summarized in Table 2.

## 4. Regulation of Polyamine Metabolism

Odc, Samdc, and Ssat are the three key enzymes governing polyamine metabolism. The two decarboxylases are the rate-limiting enzymes of polyamine biosynthesis whilst Ssat controls the polyamine interconversion cycle [33]. In some dimorphic fungi such as *Yarrowia lipolytica*, *Candida. albicans, U. maydis, Mucor rouxii*, and *Mucor circinelloides,* Odc activity is related to morphogenetic processes [16, 54–56]. In the budding yeast *S. cerevisiae*, control of Odc is under negative feedback regulation, by the end products of the pathway and the loss of enzyme activity is the result of increased degradation of Odc. Polyamines reduce the half-life of the newly synthesized Odc protein from 3 h to approximately 10 min [57]. Additionally, polyamines induce the expression of a protein inhibitor of Odc, called the antienzyme (Az) reviewed in [58]. 

 The antienzyme was first discovered in mammals, and later on shortened its name to antizyme. In vertebrates there exists a family of antizymes encoded by three genes: *AZ1, AZ2,* and *AZ3*; *AZ1* and *AZ2* are expressed in all tissues, but it seems that the role exerted by *AZ1* is predominant over *AZ2* as judged by the amount of *AZ1* mRNA. On the other hand, *AZ3* has been identified only during spermiogenesis reviewed in [59]. Nevertheless, recently it was demonstrated that *AZ3 *encodes the protein p12, which does not regulate Odc activity, instead it regulates a protein phosphatase [60]. The presence of antizyme in fungi was later on demonstrated [61, 62]. 

 As mentioned earlier, Odc is the rate-limiting enzyme during polyamine biosynthesis. In order to be active, Odc forms a homodimer having two enzymatic sites. Odc degradation proceeds through a sophisticated mechanism involving the participation of the antizyme that shows higher affinity for Odc, displacing the weakly associated Odc homodimer. After Az interacts with Odc an inactive heterodimer is formed. Odc degradation is carried out in two steps: first, the heterodimers (Odc : antizyme) are separated; the second step is the presentation of these heterodimers to the 26S proteasome [63]. During the establishment of the heterodimer, the C-terminus of the Odc protein is exposed, an indispensable phenomenon for degradation of the protein by the proteasome, being considered a destabilizing epitope that in turn is recognized by the 26 proteasome [63]. It is important to mention that Az is able to regulate uptake of polyamines by the cell as well [64]. In turn, Az is inhibited by a protein with high similarity to Odc but devoid of enzymatic activity named antizyme inhibitor [65]. It is interesting that Az, in contrast to Odc, is degraded by an ubiquitin-dependent mechanism [62].

In the kingdom Fungi the first antizyme encoding gene (*SPA*) cloned was the one from *Schizosaccharomyces pombe*. Deletion of the corresponding gene did not impair its viability but resulted in an abnormal accumulation of polyamines in the cell [61]. Accordingly, a 40-fold increase of polyamines was detected in stationary phase culture of the mutant cells, revealing that the Az is the main mechanism to control polyamine synthesis in this yeast. In contrast when *SPA* gene was overexpressed, putrescine was no longer detected [61]. In support of these data [66], it was observed that the half-life of Odc is shortened in a Δ*spe2 S. pombe *as compared to the wild-type strain. 

 In *S. cerevisiae* the ortholog gene to *AZ*, named *OAZ1,* was identified by Palanimurugan et al. [62]. In this yeast, spermidine induces Az protein levels without affecting transcription of the *AZ* gene. The synthesis of the protein is conducted through a frameshift reaction (see below). In this case, deletion of frame 2 renders mutants with no ability to degrade Odc [62]. A number of fungal antizyme genes were further identified and the sequences described for *Pneumocystsis carinii, Botryotinia fuckeliana, *and* Emericella nidulan *[61]. Nevertheless, no experimental studies have been conducted in these organisms. It was possible to identify also 11 sequences of yeast species that are closely related to the *S. cerevisiae *protein [67]. 

In yeasts, one alternative mechanism proposed to regulate polyamine biosynthesis not related with the antizyme involves the enzyme methylthioadenosine phosphorylase (Meu1); nevertheless studies conducted by [68] in mutants of a Δ*meu1* background revealed that they contained higher levels of putrescine but lower amount of spermidine as compared with the parental strain (*MEU1*). This experiment was interpreted to be due to an inhibition of Spe by the methylthioadenosine accumulated in this strain.

In yeast polyamine inhibition of polyamine synthesis by Az is a reversible process, suggested by the fact that heterodimer (Odc : Az) is inhibited when Odc is previously inactivated by DFMO [69]. Besides, this is the first report showing the 1  :  1 stoichiometric relation between Odc and Az. Additionally, the specificity of this general posttranslational mechanism must be highlighted, considering the fact that despite the high similarity between mammalian and yeast Odc enzymes, the yeast Odc enzyme was not processed by the mammalian 26 proteasome *in vitro* [70].

 When polyamine levels increase in the cell, Az is synthesized. Production of the antizyme requires a translational frameshift to align a small upstream open reading frame (ORF) with a second ORF that encodes the activated protein. This binds to Odc as described above, that is hydrolyzed by the 26S proteasome without ubiquitination.

An interesting species-specific difference in Odc is that its degradation signal (“degron”) is located at the *N*-terminus in the yeast Odc in contrast to human Odc, in which it is located at the *C*-terminus of the protein (see above). This sequence is important for Odc degradation by an independent ubiquitin mechanism. In this process intervenes an unstructured short region, which can be replaced by another unrelated but also unstructured sequence which is functional only in an alpha helix (but no with beta configuration). In the yeast, Odc degradation strictly depends on the presence of Az. Nevertheless in mammalian or yeast Odc's degradation occurs *in vitro* in the absence of Az [71]. In fact, the presence of the unstructured domain in an adequate context allows the degradation of the protein by an ubiquitin-independent mechanism [72].

A peculiar epigenetic mechanism to regulate polyamine metabolism has been described recently in *S. cerevisiae*. In this phenomenon, the misfolded terminal translational release factor eRF3T constitutes a “prion” protein, PSI+. The conformational changes of this protein impair its termination efficiency, increasinging in this way the stop codon readthrough ratio having as a consequence proteins with a longer *C* terminal extension [73]. Accordingly, expression of the full length Az (frame 1 plus frame 2) was observed in PSI+ strains, whereas it was barely detected in the *psi-*strain. As a result, Odc degradation was higher in the PSI+ strains that contained low polyamines levels, in contrast to *psi-*mutants where low Odc degradation was observed. Worth to mention is the fact that this “prion” is not present in all *S. cerevisiae* strains [73].

## 5. Transport of Polyamines in Fungi

Polyamine transport involves uptake and export mechanisms and they play an important role in the homeostatic regulation of the polyamine levels. As described above, inhibitors of polyamines biosynthesis gave a very good control of plant infections by some fungi, while some others were much less sensitive to polyamine biosynthesis inhibitors. In view of this fact, examination of the mechanisms underlying the different response was undertaken by different authors. The first report of polyamine uptake involved a plant pathogenic fungus *Fusarium culmorum, *West and Walters [74] were able to demonstrate using ^14^C-labelled polyamines that their uptake was pH dependent and biphasic. More important, they observed differences between putrescine and spermidine uptake, which led them to consider that the transport of each one of them was given by specific systems. Recently, it was demonstrated that most of the identified polyamine transport proteins are polyamine specific or polyamine preferential transport proteins [75].

 Transport of polyamines in fungi has been studied most extensively in *Neurospora crassa *and *S. cerevisiae * [76]. In *S. cerevisiae, *four genes that encode polyamine excretion proteins TPO1-TPO4 have been described. These proteins are mainly located in the plasma membrane; TPO1 and TPO4 were found to be able to transport putrescine, spermidine, and spermine, while TPO2 and TPO3 proved specific of spermine [77–79]. Also known are some other proteins which transport polyamines to the vacuole and Golgi apparatus: UGA4 and TPO5, respectively. UGA4 preferred putrescine as substrate, wheras TPO5 was able to transport both, putrescine and spermidine [80, 81]. A putative polyamine transporter with 26.1% of identity and 53.1% of similarity to UGA4 has been isolated from *C. albicans *[82], and an *in silico *search of homologous genes in the genomes of several phytopathogenic fungi revealed the presence of these polyamine transporters, suggesting the existence of a general mechanism of transport in these organisms (Table 3). However, the results do not eliminate the possibility of the existence of other eukaryotic polyamine transporters, and much work remains to be done regarding the analysis of polyamine transporters in phytophathogenic fungi.

In addition to these studies, protein kinases have been reported to increase polyamine uptake. In yeast cells it was found that Dur3, a protein that catalyzes the uptake of polyamines together with urea, was activated by phosphorylation of its Thr^250^, Ser^251^, and Thr^684^ residues by the polyamine transport protein kinase 2 [83]. This result suggests that the inability to produce spermidine by pathogenic mutants deficient in its synthesis, may be at least in part, compensated by polyamine uptake from the host. Therefore, host mutants affected in polyamine transport may be at least partially resistant to fungal pathogens, indicating that inhibition of polyamine transporters may decrease or completely avoid their virulence. The use of microarrays to compare mutants and wild type strains may be useful in the identification of putative polyamine transporters.

 Originally, it was considered that inhibition of polyamine synthesis would be enough to control the growing of some phytophathogenic fungi. However, as already indicated, the failure of the treatments may be due to an increase in the uptake of host polyamines by the pathogens. Nevertheless, what we know about polyamine transporters leads us to consider the possibility of combining inhibition of polyamine biosynthesis together with inhibition of plant polyamine transporters, as a way to avoid this secondary effect. In this way, phytopathogenic fungi would not be able to survive by taking polyamines from the host. To fulfill this objective, a better characterization of the polyamine transport proteins in both fungi and plants is essential. Knowledge and better characterization of these mechanisms may facilitate the utilization of inhibitors of polyamine uptake and secretion in the design of novel strategies of biological control.

## 6. Polyamines in Growth, Cell Differentiation, and Morphogenesis of Fungi

Polyamines are essential for growth as has been extensively reconfirmed in several fungus systems such as *Neurospora crassa, S. cerevisiae, T. yallundae, Yarrowia lipolytica, and U. maydis. *Additionally, polyamines have been implicated in the regulation of both cell proliferation and differentiation, and it has been demonstrated in different systems that high concentrations of polyamines may be required for the operation of differentiation phenomena in fungi [16, 17, 84–87]. Accordingly, several studies have demonstrated that changes in polyamine metabolism precede a wide variety of morphogenetic events. Thus, as reviewed by [6], specific inhibition of putrescine biosynthesis by addition of the Odc inhibitor DAB, at concentrations that did not affect vegetative growth, specifically inhibited differentiation phenomena such as spore germination, sporulation, and dimorphic transition in different fungi. Addition of exogenous putrescine in all cases reverted the action of the drug. Other well-studied differentiation events affected by the inhibition of polyamine biosynthesis are, appressorium development, germination, and filament formation [88–90].

 It has been described also that *odc* mutants of dimorphic fungi such as *U. maydis* [16, 88] and* Yarrowia lipolytica* [87] are unable to carry out the dimorphic transition when incubated with limiting concentrations of putrescine that do not inhibit their growth. However, the precise biological mechanism by which polyamines affect these processes remains uncertain.

 Taking into consideration that *odc* mutants are unable to synthesize any of the cell polyamines, it remained unclear which of them was the most important one involved in the morphogenetic process. To answer this question we turned to the use of *U. maydis* that, as indicated above, contains only putrescine and spermidine, but not spermine. For these experiments a mutant unable to synthesize putrescine by neither the biosynthetic nor degradative mechanism of *U. maydis* carrying mutations in the genes encoding Odc and Pao (*odc/pao *double mutants) was used. It was found that this mutant was able to grow and carry out the dimorphic transition with the addition of spermidine only, that is, in the complete absence of putrescine [30]. This result evidenced that spermidine is the polyamine involved in practically all the functions of polyamines in this, and probably all fungi. In agreement with this result, other authors have hypothesized that spermidine might be specifically required for cell differentiation [91, 92].

## 7. Behavior of Fungal Mutants Affected in Genes Encoding Enzymes of the Polyamine Pathway

Studies on mutants affected in the synthesis of polyamines in fungal plant pathogens are scarce. *odc* mutants have been generated in the wheat pathogens *Stagonospora nodorum *and *Tapesia yallundae* and in the corn pathogen *U. maydis*. In either case impairment of polyamine synthesis by *odc* disruption rendered auxotrophic mutants, which were able to grow only when polyamines, either putrescine or spermidine, were added to the culture media, confirming in this way the existence of a unique pathway for the synthesis of polyamines [16, 30, 55, 86, 87, 93].

 Regarding the pathogenicity of the mutants, *S. nodorum odc* showed to be less virulent than the wild-type strain. In contrast *T. yallundae odc *mutants required polyamines to form the infective structures and the typical branched mycelium *in vitro, *but the pathogenicity test revealed no difference between the wild type strain and the mutants, and in fact similar amounts of biomass were formed by *T. yallundae odc* mutants as compared to the wild type. Possibly this difference is due, as occurred in the case of the addition of inhibitors, to the ability of the mutants to uptake the free polyamines present in the plant, considering that a 50 *μ*M concentration of exogenous polyamines was enough to satisfy the auxotrophic phenotype observed *in vitro* [86]. The observation that two wheat pathogens impaired in polyamines synthesis behaved very different when they are in contact with the plant host opens the opportunity to conduct more comprehensive studies in different pathosystems involving mutants affected in different genes of the polyamine metabolism. In the case of *S. nodorum* it is worth to considerer polyamines as a promising target to design new fungicides [6]. 

 To our knowledge, the study of polyamine synthesis at the genetic level on a phytopathogenic fungus has been conducted at a deeper detail level in *U. maydis *[16, 30, 64, 91]. *U. maydis,* the causal agent of common smut in maize, is considered a model for the study of fungal pathogenicity and cellular differentiation. Mixtures of sexually compatible polyamine auxotrophic mutants affected in the *ODC *gene inoculated in maize were unable to cause any disease symptom in the plants. In contrast, when the same experiment was carried out with *U. maydis *mutants affected in the gene encoding *Spe*, it was observed that only 20% of the inoculated plants were infected at a similar rate as the plants inoculated with the wild type crosses [87]. Interestingly, mutants affected in the *SAMDC *gene (the other gene required for the synthesis of spermidine) were completely avirulent to maize. However, a more important polyamine role in virulence could be learned by the fact that although *pao *single mutants were not auxotrophic to polyamines, they presented a reduced virulence [91]. These results were explained on the bases that mutants were able or not to import polyamines from the host and reveal that fungi have different requirements for polyamines, as well as different capacities to obtain polyamines from their hosts and develop their pathogenesis processes.

## 8. Polyamines in Host-Fungus Interaction

We need to stress that the rate of polyamine synthesis depends on the pathosystem under study. In the case of barley, when challenged with rust or powdery mildew or *M. grisea*-rice, a clear increase in the amount of polyamines has been detected. Contrary to this result, when tobacco plants were challenged with powdery mildew or downy mildew the polyamine levels were dramatically reduced [94]. In another work, the levels of conjugated forms of putrescine and spermidine decreased, while the conjugated form of spermine increased, when sugarcane was infected with *Ustilago scitaminea *[95]. In the same way, *U. maydis *induces alteration in polyamine metabolism of maize tumors, with an increase of putrescine, correlated with the activation of *ADC, SAMDC1, ZMSAMDC2*, and * ZMSAMDC3* gene expression [96, 97].

Recently it was observed that tomato transgenic plants expressing the yeast Spe accumulate higher levels of polyamines than the wild type strain. These transgenic lines were more susceptible to the attack by *B. cinerea*, but not by *Alternaria solani* [98]. Addition of polyamine inhibitors (DFMO or CHA) restores the normal response of the transgenic leaves when challenged with *B. cinerea*. In contrast, exogenous spermidine increases the susceptibility of wild type leaves to the pathogen. These studies demonstrate the importance to keep the homeostasis of polyamines, revealing that imbalance of spermidine impairs host signaling cascades leading to alterations in the immune responses against *B. cinerea* but not against *A. solani* [98].

A notable example of the role of polyamines in plant infection by fungi is the case of the fusarium head blight, a disease produced in wheat and other small grain cereals by *Fusarium* species [99]. Production of the trichothecene mycotoxin deoxynivalenol (DON) by the fungus in the infected heads is required for full virulence, since the degree of virulence of *F. graminearum* is directly related to the amounts of DON produced, and it appears to be indispensable for the pathogen to spread from one infected head to another one [100]. This metabolite is not only phytotoxic but it also constitutes a significant threat to human and animal health [101, 102]. Interestingly, DON's synthesis is induced by a wide variety of plant compounds including arginine, ornithine, agmatine, citrulline, and putrescine, all of them natural precursors of the polyamines synthesis pathway [103]. It has been hypothesized that the pathogen senses the polyamines as a signal to trigger the production of these toxins necessary for the establishment of the infection [100]. *In vitro*, the synthesis of DON is regulated at the transcriptional level by the addition of some polyamines in the culture media, and the amount produced was similar to that quantified in the plants infected with *F. graminearum* [100]. Until now, it has not been possible to determine the origin of the DON-inducing polyamines, whether from the plant or the fungal pathogen, although the detection of putrescine levels matches with the levels of expression of the genes involved in polyamine biosynthesis from the plant [103]. Further research using *F. graminearum* mutants impaired in the synthesis of polyamines would be very helpful to understand the phenomenon.

## 9. Future Perspectives

From all the aspects discussed above, it is clear that polyamine metabolism is an important factor in the relationship existing between a pathogen and its host, a factor that alternatively may incline the balance in favor of one or the other. In this sense, fungi are good models to understand the role of polyamines in pathogenesis. The complexity of polyamine metabolism is such that it involves synthesis, degradation, and transport. As we have considered, there is the need of a closer examination of transport proteins involved in the intake and export of polyamines in phytopathogenic fungi. Each one of these aspects by itself or the sum of them may be essential to overcome polyamine deficiencies in the pathogen or the host, changing the final outcome of the infection. This is an interesting aspect that may lead to the design of inhibitors and strategies directed to specific targets of the pathogen for the treatment and prevention of important plant diseases responsible of severe economical losses and hunger worldwide.

## Figures and Tables

**Table 1:** Distribution of enzymes involved in polyamine biosynthesis in phytophatogenic fungi and fungal-related Chromista organisms. We used *S. cerevisiae *well-characterized proteins as a template for *in silico* searches. The access codes of the Genbank entries are shown. (—) indicates absence of an homologous gene.

Phytopapthogenic fungi, or fungal-related organisms	Phylum	Disease	Ornithine decarboxylase	Spermidine synthase	Spermine synthase	Polyamine oxidase
*Sclerotinia sclerotiorum*	Ascomycota	Multihost rot diseases	*SS1G_05468.3*	*SS1G_10282*	*SS1G_12906.3*	*SS1G_00866.3*
*Fusarium oxysporum 4287*	Ascomycota	Multihost wilt disease	*FOXG_07603.2*	*FOXG_05165.2*	*—*	*FOXG_17483.2*
*Fusarium verticillioides 7600*	Ascomycota	Maize seed rot	*FVEG_04532.3*	*FVEG_02968.3*	*—*	*FVEG_03013.3*
*Fusarium graminearum PH-1*	Ascomycota	Wheat/barley head blight	*FGSG_05903.3*	*FGSG_13725.3*	*—*	*FGSG_10284.3*
*Magnaporthe_grisea*	Ascomycota	Rice blast disease	*MGG_02441.6*	*MGG_06197.6*	*MGG_01296.6*	*MGG_13246.6*
*Nectria hematococa v 2.0*	Ascomycota	Pea wilt	*63797*	*40897*	*32447*	*100632*
*Mycosphaerella fijiensis*	Ascomycota	Banana black leaf streak	*31208*	*27743*	*—*	*76981*
*Mycosphaerella graminicola*	Ascomycota	Wheat leaf blotch	*63580*	*45927*	*2056*	*71007*
*Stagonospora_nodorum*	Ascomycota	Wheat glume blotch	*SNOG_09474*	*SNOG_10727*	*SNOG_09768*	*SNOG_10520*
*Puccinia graminis tritici*	Basidiomycota	Cereal rusts	*PGTG_12488.4*	*PGTG_06954.4*	*—*	*PGTG_14428.4*
*Puccinia triticina*	Basidiomycota	Cereal rusts	*PTTG_05519.1*	*PTTG_07923.1*	*—*	*—*
*Ustilago maydis*	Basidiomycota	Corn smut disease	*Um01048.1*	*Um05818*	*—*	*Um05850*
*Phytophthora infestans*	Stramenopiles	Late blight potato blight	*PITG_02303.1*	*PITG_10866.2*	*PITG_11271.2*	*PITG_04877.1*
*Phytophthora ramorum*	Stramenopiles	Sudden oak death	*49709*	*72107*	*—*	*79768*
*Phytophthora sojae*	Stramenopiles	Soybean stem/root rot	*108272*	*109524*	*133564*	*129047*

**Table 2:** Effect of polyamine inhibitors on fungal growth.

Fungus	DFMO	DFMA	MGBG	Reference
*C. gloesporioides*			+	[47]
*S. tritici*	−			[41]
*U. maydis*	−			[41]
*S. nodorum*	−			[52]
*P. avenae*	−			[52]
*O. ulmi*	−			[52]
*S. sclerotiorum*	+	+		[42, 48]
*P. sojae*	+			[48]
*C. truncatum*	+			[42]
*R. solani*	+			[40]
*B. cinerea*	+			[43–45]
*F. oxysporum*	+			[43–45]
*C. carbonum*	+			[43–45]
*P. infestans*	+			[40]
*B. cinerea*		+		[40]
*P. infestans*		+		[40]

+: growth inhibition; −: no inhibition of growth. DFMO: D, L-*α*-difluoromethylornithine, ornithine decarboxylase inhibitor. DFMA: difluoromethylarginine, arginine decarboxylase inhibitor. MGBG: methyglyoxal bis-[guanyl hydrazone], adenosylmethionine decarboxylase inhibitor.

**Table 3:** Distribution of proteins involved in polyamine transport in phytophatogenic fungi. We used *S. cerevisiae *well-characterized proteins as a template for *in silico* searches. The access codes of the Genebank entries are shown.

Phytopapthogenic fungus	Disease	TPO1	TPO2	TPO3	TPO4	TPO5	UGA4	DUR3	SAM3	SKY1
*Sclerotinia sclerotiorum*	*Multihost rot diseases*	*SS1G-02048.3*	*SS1G_11948.3*	*SS1G_11948.3*	*SS1G_07364.3*	*SS1G_07859.3*	*SS1G_04877*	*SS1G_06216.3*	*SS1G_05757.3*	*SS1G_03277.3*
*Fusarium oxysporum* 4287	*Multi-host wilt disease*	*FOXG_13864.2*	*FOXG_08371.2*	*FOXG_08371.2*	*FOXG_09760.2*	*FOXG_10394.2*	*FOXG_02474*	*FOXG_12291.2*	*FOXG_00434.2*	*FOXG_15645.2*
*Fusarium verticillioides* 7600	*Maize seed rot*	*FVEG_11294.2*	*FVEG_06303.3*	*FVEG_06303.0*	*FVEG_08722.3*	*FVEG_09045.3*	*FVEG_05661.3*	*FVEG_10834.3*	*FVEG_01077.3*	*FVEG_13105.3*
*Fusarium graminearum* PH-1	*Wheat/barley head blight*	*FSGS_04370.3*	*FGSG_09697.3*	*FGSG_09697.3*	*FGSG_03725.3*	*FSGS_02439.3*	*FSGS_06473.3*	*FSGS_03111.3*	*FSGS_00613.3*	*FSGS_02795.3*
*Magnaporthe_grisea*	*Rice blast disease*	*MGG_03205.6*	*MGG_04158.6*	*MGG_04158.6*	*MGG_03205.6*	*MGG_14115.6*	*MGG_01385.6*	*MGG_04385.6*	*MGG_11062.6*	*MGG_10596.6*
*Nectria hematococav* 2.0	*Pea wilt*	*101392*	*57916*	*57916*	*87421*	*104730*	*34803*	*70607*	*46621*	*92214*
*Mycosphaerella fijiensis*	*Banana black leaf streak*	*27972*	*28889*	*45287*	*27972*	*45015*	*36373*	*84945*	*12171*	*2883*
*Mycosphaerella graminicola*	*Wheat leaf blotch*	*48906*	*50337*	*50337*	*52173*	*3259*	*38643*	*75866*	*63940*	*13647*
*Stagonospora nodorum*	*Wheat glume blotch*	*SNOG_01606*	*SNOG_02773*	*SNOG_02773*	*SNOG_06724*	*SNOG_01622*	*SNOG_07765*	*SNOG_02631*	*SNOG_03538*	*SNOG_08074*
*Phytophthora infestans*	*Late blight potato blight*	*—*	*PITG_11748.2*	*PITG_11748.2*	*PITG_13000.2*	*PITG_00228.1*	*PITG_11471.2*	*—*	*—*	*PITG_00407.1*
*Phytophthora ramorum*	Sudden oak death	—	*84883*	*73291*	*84885*	*72585*	*73775*	—	—	38571
*Phytophthora sojae*	Soybean stem/root rot	—	108536	108536	131465	156002	130593	—	—	112647
*Ustilago maydis*	Corn smut diseases	*Um02723.2*	*Um01996*	*Um05248*	*Um02723.2*	*Um02146*	*Um03522*	*Um04577*	*Um00343*	*Um03796*
*Puccinia graminis tritici*	Cereal rusts	*PGTG_06694.4*	*PGTG_06694.4*	*PGTG_06694.4*	*PGTG_06694.4*	*PGTG_03181.4*	*PGTG_03181.4*	*—*	*PGTG_10749.4*	*PGTG_15313.4*
*Puccinia triticina*	Cereal rusts	*PTTG_07888.1*	*PTTG_07888.1*	*PTTG_07888.1*	*PTTG_07888.1*	*PTTG_02870*	*PTTG_02870*	*—*	*PTTG_04646.1*	*PTTG_03241.1*

## References

[1] Tabor CW, Tabor H (1983). Polyamines. *Annual Review of Biochemistry*.

[2] Pegg AE (2009). Mammalian polyamine metabolism and function. *IUBMB Life*.

[3] Igarashi K, Kashiwagi K (2010). Characteristics of cellular polyamine transport in prokaryotes and eukaryotes. *Plant Physiology and Biochemistry*.

[4] Hu RH, Pegg AE (1997). Rapid induction of apoptosis by deregulated uptake of polyamine analogues. *Biochemical Journal*.

[5] Madeo F, Eisenberg T, Büttner S, Ruckenstuhl C, Kroemer G (2010). Spermidine: a novel autophagy inducer and longevity elixir. *Autophagy*.

[6] Ruiz-Herrera J (1994). Polyamines, DNA methylation, and fungal differentiation. *Critical Reviews in Microbiology*.

[7] Khurana N, Saxena RK, Gupta R, Rajam MV (1996). Polyamines as modulators of microcycle conidiation in *Aspergillus flavus*. *Microbiology*.

[8] Ouameur AA, Tajmir-Riahi HA (2004). Structural analysis of DNA interactions with biogenic polyamines and cobalt(III)hexamine studied by fourier transform infrared and capillary electrophoresis. *Journal of Biological Chemistry*.

[9] D’Agostino L, Di Pietro M, Di Luccia A (2005). Nuclear aggregates of polyamines are supramolecular structures that play a crucial role in genomic DNA protection and conformation. *FEBS Journal*.

[10] Kusano T, Berberich T, Tateda C, Takahashi Y (2008). Polyamines: essential factors for growth and survival. *Planta*.

[11] Banuett F (1995). Genetics of *Ustilago maydis*, a fungal pathogen that induces tumors in maize. *Annual Review of Genetics*.

[12] Tsukuda T, Carleton S, Fotheringham S, Holloman WK (1988). Isolation and characterization of an autonomously replicating sequence from *Ustilago maydis*. *Molecular and Cellular Biology*.

[13] Fotheringham S, Holloman WK (1990). Pathways of transformation in *Ustilago maydis* determined by DNA conformation. *Genetics*.

[14] Bölker M (2001). *Ustilago maydis*—a valuable model system for the study of fungal dimorphism and virulence. *Microbiology*.

[15] Basse CW, Steinberg G (2004). *Ustilago maydis*, model system for analysis of the molecular basis of fungal pathogenicity. *Molecular Plant Pathology*.

[16] Guevara-Olvera L, Xoconostle-Cázares B, Ruiz-Herrera J (1997). Cloning and disruption of the ornithine decarboxylase gene of *Ustilago maydis*: evidence for a role of polyamines in its dimorphic transition. *Microbiology*.

[17] Valdés-Santiago L, Cervantes-Chávez JA, Ruiz-Herrera J (2009). *Ustilago maydis* spermidine synthase is encoded by a chimeric gene, required for morphogenesis, and indispensable for survival in the host. *FEMS Yeast Research*.

[18] Casero RA, Pegg AE (1993). Spermidine/spermine N1-acetyltransferase—the turning point in polyamine metabolism. *FASEB Journal*.

[19] Casero RA, Marton LJ (2007). Targeting polyamine metabolism and function in cancer and other hyperproliferative diseases. *Nature Reviews Drug Discovery*.

[20] Vujcic S, Halmekytö M, Diegelman P (2000). Effects of conditional overexpression of spermidine/spermine N1-acetyltransferase on polyamine pool dynamics, cell growth, and sensitivity to polyamine analogs. *Journal of Biological Chemistry*.

[21] Bazzicalupi C, Bencini A, Cohen H (1998). Palladium(II) co-ordination by linear N-methylated polyamines: a solution and solid-state study. *Journal of the Chemical Society*.

[22] Pegg AE, McCann PP (1982). Polyamine metabolism and function. *The American Journal of Physiology*.

[23] Wang Y, Devereux W, Woster PM, Stewart TM, Hacker A, Casero RA (2001). Cloning and characterization of a human polyamine oxidase that is inducible by polyamine analogue exposure. *Cancer Research*.

[24] Vujcic S, Diegelman P, Bacchi CJ, Kramer DL, Porter CW (2002). Identification and characterization of a novel flavin-containing spermine oxidase of mammalian cell origin. *Biochemical Journal*.

[25] Cona A, Cenci F, Cervelli M (2003). Polyamine oxidase, a hydrogen peroxide-producing enzyme, is up-regulated by light and down-regulated by auxin in the outer tissues of the maize mesocotyl. *Plant Physiology*.

[26] Alcázar R, Marco F, Cuevas JC (2006). Involvement of polyamines in plant response to abiotic stress. *Biotechnology Letters*.

[27] Tavladoraki P, Rossi MN, Saccuti G (2006). Heterologous expression and biochemical characterization of a polyamine oxidase from Arabidopsis involved in polyamine back conversion. *Plant Physiology*.

[28] Moschou PN, Sanmartin M, Andriopoulou AH, Rojo E, Sanchez-Serrano JJ, Roubelakis-Angelakis KA (2008). Bridging the gap between plant and mammalian polyamine catabolism: a novel peroxisomal polyamine oxidase responsible for a full back-conversion pathway in arabidopsis. *Plant Physiology*.

[29] Landry J, Sternglanz R (2003). Yeast Fms1 is a FAD-utilizing polyamine oxidase. *Biochemical and Biophysical Research Communications*.

[30] Valdés-Santiago L, Guzmán-De-Peña D, Ruiz-Herrera J (2010). Life without putrescine: disruption of the gene-encoding polyamine oxidase in *Ustilago maydis* odc mutants. *FEMS Yeast Research*.

[31] Seiler N (1980). On the role of GABA in vertebrate polyamine metabolism. *Physiological Chemistry and Physics*.

[32] Angelini R, Cona A, Federico R, Fincato P, Tavladoraki P, Tisi A (2010). Plant amine oxidases “on the move”: an update. *Plant Physiology and Biochemistry*.

[33] Urdiales JL, Medina MÁ, Sánchez-Jiménez F (2001). Polyamine metabolism revisited. *European Journal of Gastroenterology and Hepatology*.

[34] Nickerson KW, Dunkle LD, Van Etten JL (1977). Absence of spermine in filamentous fungi. *Journal of Bacteriology*.

[35] Pegg AE, Michael AJ (2010). Spermine synthase. *Cellular and Molecular Life Sciences*.

[36] Watson MB, Emory KK, Piatak RM, Malmberg RL (1998). Arginine decarboxylase (polyamine synthesis) mutants of *Arabidopsis thaliana* exhibit altered root growth. *Plant Journal*.

[37] Aveyard P, Adab P, Cheng KK, Wallace DMA, Hey K, Murphy MFG (2002). Does smoking status influence the prognosis of bladder cancer? A systematic review. *British Journal of Urology International*.

[38] Slocum RD, Galston AW (1985). In vivo inhibition of polyamine biosynthesis and growth in tobacco ovary tissues. *Plant and Cell Physiology*.

[39] Pieckenstain FL, Gárriz A, Chornomaz EM, Sánchez DH, Ruiz OA (2001). The effect of polyamine biosynthesis inhibition on growth and differentiation of the phytopathogenic fungus *Sclerotinia sclerotiorum*. *Antonie van Leeuwenhoek*.

[40] Walters DR (1995). Inhibition of polyamine biosynthesis in fungi. *Mycological Research*.

[41] Smith TA, Barker JHA, Jung M (1990). Growth inhibition of *Botrytis cinerea* by compounds interfering with polyamine metabolism. *Journal of General Microbiology*.

[42] Gamarnik A, Frydman RB, Barreto D (1994). Prevention of infection of soybean seeds by *Colletotrichum truncatum* by polyamine biosynthesis inhibitors. *Phytopathology*.

[43] Rajam MV, Galston AW (1985). The effects of some polyamine biosynthetic inhibitors on growth and morphology of phytopathogenic fungi. *Plant and Cell Physiology*.

[44] West HM, Walters DR (1989). Effects of polyamine biosynthesis inhibitors on growth of pyrenophora-teres, gaeumannomyces-graminis, fusarium-culmorum and septoria-nodorum in vitro. *Mycological Research*.

[45] Mackintosh CA, Walters DR (1997). Growth and polyamine metabolism in *Pyrenophora avenae* exposed to cyclohexylamine and norspermidine. *Amino Acids*.

[46] Gárriz A, Dalmasso MC, Pieckenstain FL, Ruiz OA (2003). The putrescine analogue 1-aminooxy-3-aminopropane perturbs polyamine metabolism in the phytopathogenic fungus *Sclerotinia sclerotiorum*. *Archives of Microbiology*.

[47] Ahn IP, Kim S, Choi WB, Lee YH (2003). Calcium restores prepenetration morphogenesis abolished by polyamines in *Colletotrichum gloeosporioides* infecting red pepper. *FEMS Microbiology Letters*.

[48] Chibucos MC, Morris PF (2006). Levels of polyamines and kinetic characterization of their uptake in the soybean pathogen *Phytophthora sojae*. *Applied and Environmental Microbiology*.

[49] Choi WB, Kang SH, Lee YW, Lee YH (1998). Cyclic AMP restores appressorium formation inhibited by polyamines in *Magnaporthe grisea*. *Phytopathology*.

[50] Kumar A, Altabella T, Taylor MA, Tiburcio AF (1997). Recent advances in polyamine research. *Trends in Plant Science*.

[51] Smith TA, Barker JHA, Owen WJ (1992). Insensitivity of septoria-tritici and ustilago-maydis to inhibitors of ornithine decarboxylase. *Mycological Research*.

[52] Bailey A, Mueller E, Bowyer P (2000). Ornithine decarboxylase of *Stagonospora (Septoria) nodorum* is required for virulence toward wheat. *Journal of Biological Chemistry*.

[53] Khan AJ, Minocha SC (1989). Biosynthetic arginine decarboxylase in phytopathogenic fungi. *Life Sciences*.

[54] Guevara-Olvera L, Calvo-Mendez C, Ruiz-Herrera J (1993). The role of polyamine metabolism in dimorphism of *Yarrowia lipolytica*. *Journal of General Microbiology*.

[55] Bailey A, Mueller E, Bowyer P (2000). Ornithine decarboxylase of *Stagonospora (Septoria) nodorum* is required for virulence toward wheat. *Journal of Biological Chemistry*.

[56] Blasco JL, García-Sánchez MA, Ruiz-Herrera J, Eslava AP, Iturriaga EA (2002). A gene coding for ornithine decarboxylase (odcA) is differentially expressed during the *Mucor circinelloides* yeast-to-hypha transition. *Research in Microbiology*.

[57] Toth C, Coffino P (1999). Regulated degradation of yeast ornithine decarboxylase. *Journal of Biological Chemistry*.

[58] Coffino P (2001). Regulation of cellular polyamines by antizyme. *Nature Reviews Molecular Cell Biology*.

[59] Chen H, MacDonald A, Coffino P (2002). Structural elements of antizymes 1 and 2 are required for proteasomal degradation of ornithine decarboxylase. *Journal of Biological Chemistry*.

[60] Ruan Y, Cheng M, Ou Y, Oko R, Van Der Hoorn FA (2011). Ornithine decarboxylase antizyme Oaz3 modulates protein phosphatase activity. *Journal of Biological Chemistry*.

[61] Ivanov IP, Matsufuji S, Murakami Y, Gesteland RF, Atkins JF (2000). Conservation of polyamine regulation by translational frameshifting from yeast to mammals. *EMBO Journal*.

[62] Palanimurugan R, Scheel H, Hofmann K, Dohmen RJ (2004). Polyamines regulate their synthesis by inducing expression and blocking degradation of ODC antizyme. *EMBO Journal*.

[63] Zhang M, Pickart CM, Coffino P (2003). Determinants of proteasome recognition of ornithine decarboxylase, a ubiquitin-independent substrate. *EMBO Journal*.

[64] Belting M, Mani K, Jönsson M (2003). Glypican-1 is a vehicle for polyamine uptake in mammalian cells: a pivotal role for nitrosothiol-derived nitric oxide. *Journal of Biological Chemistry*.

[65] Sayers TJ, Brooks AD, Koh CY (2003). The proteasome inhibitor PS-341 sensitizes neoplastic cells to TRAIL-mediated apoptosis by reducing levels of c-FLIP. *Blood*.

[66] Chattopadhyay MK, Murakami Y, Matsufuji S (2001). Antizyme regulates the degradation of ornithine decarboxylase in fission yeast *Schizosaccharomyces pombe*. Study in the spe2 knockout strains. *Journal of Biological Chemistry*.

[67] Ivanov IP, Gesteland RF, Atkins JF (2006). Evolutionary specialization of recoding: frameshifting in the expression of *S. cerevisiae* antizyme mRNA is via an atypical antizyme shift site but is still +1. *RNA*.

[68] Chattopadhyay MK, Tabor CW, Tabor H (2005). Studies on the regulation of ornithine decarboxylase in yeast: effect of deletion in the MEU1 gene. *Proceedings of the National Academy of Sciences of the United States of America*.

[69] Chattopadhyay MK, Fernandez C, Sharma D, McPhie P, Masison DC (2011). Yeast ornithine decarboxylase and antizyme form a 1:1 complex in vitro: purification and characterization of the inhibitory complex. *Biochemical and Biophysical Research Communications*.

[70] Porat Z, Landau G, Bercovich Z, Krutauz D, Glickman M, Kahana C (2008). Yeast antizyme mediates degradation of yeast ornithine decarboxylase by yeast but not by mammalian proteasome: new insights on yeast antizyme. *Journal of Biological Chemistry*.

[71] Takeuchi J, Chen H, Hoyt MA, Coffino P (2008). Structural elements of the ubiquitin-independent proteasome degron of ornithine decarboxylase. *Biochemical Journal*.

[72] Gödderz D, Schäfer E, Palanimurugan R, Dohmen RJ (2011). The N-terminal unstructured domain of yeast odc functions as a transplantable and replaceable ubiquitin-independent degron. *Journal of Molecular Biology*.

[73] Namy O, Galopier A, Martini C, Matsufuji S, Fabret C, Rousset JP (2008). Epigenetic control of polyamines by the prion [PSI+]. *Nature Cell Biology*.

[74] West HM, Walters DR (1991). Polyamine uptake by the plant pathogenic fungus, fusarium-culmorum. *Mycological Research*.

[75] Kashiwagi K, Igarashi K (2011). Identification and assays of polyamine transport systems in *Escherichia coli* and *Saccharomyces cerevisiae*. *Methods in Molecular Biology*.

[76] Hoyt MA, Davis RH, Brambl R, Marzluf GA (2004). Polyamines in fungi. *The Mycota, Vol III: Biochemistry and Molecular Biology*.

[77] Tomitori H, Kashiwagi K, Sakata K, Kakinuma Y, Igarashi K (1999). Identification of a gene for a polyamine transport protein in yeast. *Journal of Biological Chemistry*.

[78] Tomitori H, Kashiwagi K, Asakawa T, Kakinuma Y, Michael AJ, Igarashi K (2001). Multiple polyamine transport systems on the vacuolar membrane in yeast. *Biochemical Journal*.

[79] Uemura T, Kashiwagi K, Igarashi K (2005). Uptake of putrescine and spermidine by Gap1p on the plasma membrane in *Saccharomyces cerevisiae*. *Biochemical and Biophysical Research Communications*.

[80] Uemura T, Tomonari Y, Kashiwagi K, Igarashi K (2004). Uptake of GABA and putrescine by UGA4 on the vacuolar membrane in *Saccharomyces cerevisiae*. *Biochemical and Biophysical Research Communications*.

[81] Tachihara K, Uemura T, Kashiwagi K, Igarashi K (2005). Excretion of putrescine and spermidine by the protein encoded by YKL174c (TPO5) in *Saccharomyces cerevisiae*. *Journal of Biological Chemistry*.

[82] McNemar MD, Gorman JA, Buckley HR (2001). Isolation of a gene encoding a putative polyamine transporter from *Candida albicans*, GPTI. *Yeast*.

[83] Uemura T, Kashiwagi K, Igarashi K (2007). Polyamine uptake by DUR3 and SAM3 in *Saccharomyces cerevisiae*. *Journal of Biological Chemistry*.

[84] Paulus TJ, Kiyono P, Davis RH (1982). Polyamine-deficient *Neurospora crassa* mutants and synthesis of cadaverine. *Journal of Bacteriology*.

[85] Balasundaram D, Tabor CW, Tabor H (1993). Oxygen toxicity in a polyamine-depleted spe2Δ mutant of *Saccharomyces cerevisiae*. *Proceedings of the National Academy of Sciences of the United States of America*.

[86] Mueller E, Bailey A, Corran A, Michael AJ, Bowyer P (2001). Ornithine decarboxylase knockout in *Tapesia yallundae* abolishes infection plaque formation in vitro but does not reduce virulence toward wheat. *Molecular Plant-Microbe Interactions*.

[87] Jiménez-Bremont JF, Ruiz-Herrera J, Dominguez A (2001). Disruption of gene YlODC reveals absolute requirement of polyamines for mycelial development in *Yarrowia lipolytica*. *FEMS Yeast Research*.

[88] Herrero AB, López MC, García S (1999). Control of filament formation in *Candida albicans* by polyamine levels. *Infection and Immunity*.

[89] Reitz M, Walters D, Moerschbacher B (1995). Germination and appressorial formation by uredospores of Uromyces viciae-fabae exposed to inhibitors of polyamine biosynthesis. *European Journal of Plant Pathology*.

[90] Rajam MV, Weinstein LH, Galston AW (1986). Kinetic studies on the control of the bean rust fungus (*Uromyces phaseoli* L.) by an inhibitor of polyamine biosynthesis. *Plant physiology*.

[91] Vuohelainen S, Pirinen E, Cerrada-Gimenez M (2010). Spermidine is indispensable in differentiation of 3T3-L1 fibroblasts to adipocytes. *Journal of Cellular and Molecular Medicine*.

[92] Deeb F, van der Weele CM, Wolniak SM (2010). Spermidine is a morphogenetic determinant for cell fate specification in the male gametophyte of the water fern *Marsilea vestita*. *Plant Cell*.

[93] Valdés-Santiago L, Cervantes-Chávez JA, Winkler R, Ruiz-Herrera J, Ruiz-Herrera J (2012). Phenotypic comparison of samdc and spe mutants reveals complex relationships of polyamine metabolism in *Ustilago maydis*. *Microbiology*.

[94] Edreva A (1997). Tobacco polyamines as affected by stresses induced by different pathogens. *Biologia Plantarum*.

[95] Legaz ME, De Armas R, Piñón D, Vicente C (1998). Relationships between phenolics-conjugated polyamines and sensitivity of sugarcane to smut (*Ustilago scitaminea*). *Journal of Experimental Botany*.

[96] Rodríguez-Kessler M, Ruiz OA, Maiale S, Ruiz-Herrera J, Jiménez-Bremont JF (2008). Polyamine metabolism in maize tumors induced by *Ustilago maydis*. *Plant Physiology and Biochemistry*.

[97] Rodriguez-Kessler M, Jimenez-Bremont JF (2009). *Ustilago maydis* induced accumulation of putrescine in maize leaves. *Plant Signaling and Behavior*.

[98] Nambeesan S, AbuQamar S, Laluk K (2012). Polyamines attenuate ethylene-mediated defense responses to abrogate resistance to *Botrytis cinerea* in tomato. *Plant Physiology*.

[99] Goswami RS, Kistler HC (2004). Heading for disaster: *Fusarium graminearum* on cereal crops. *Molecular Plant Pathology*.

[100] Gardiner DM, Kazan K, Manners JM (2009). Novel genes of *Fusarium graminearum* that negatively regulate deoxynivalenol production and virulence. *Molecular Plant-Microbe Interactions*.

[101] Ilgen P, Maier FJ, Schäfer W (2008). Trichothecenes and lipases are host-induced and secreted virulence factors of *Fusarium graminearum*. *Cereal Research Communications*.

[102] Jansen C, Von Wettstein D, Schäfer W, Kogel KH, Felk A, Maier FJ (2005). Infection pattern in barley and wheat spikes inoculated with wild-type and trichodiene synthase gene disrupted *Fusarium graminearum*. *Proceedings of the National Academy of Sciences of the United States of America*.

[103] Gardiner DM, Kazan K, Praud S, Torney FJ, Rusu A, Manners JM (2010). Early activation of wheat polyamine biosynthesis during Fusarium head blight implicates putrescine as an inducer of trichothecene mycotoxin production. *BMC Plant Biology*.

